# Hardship financing, productivity loss, and the economic cost of illness and injury in Cambodia

**DOI:** 10.1186/s12939-023-02016-z

**Published:** 2023-10-07

**Authors:** Robert John Kolesar, Guido Erreygers, Wim Van Damme, Vanara Chea, Theany Choeurng, Soklong Leng

**Affiliations:** 1 Abt Associates, Phnom Penh, Cambodia; 2 Faculty of Business and Economics, University of Antwerp, Antwerp, Belgium; 3 General Secretariat for the National Social Protection Council, Cambodian Ministry of Economy and Finance, Phnom Penh, Cambodia; 4 Centre d’Etudes et de Recherches sur le Développement International (CERDI), Université Clermont Auvergne, Clermont-Ferrand, France; 5 Centre for Health Policy, University of Melbourne, Melbourne, Australia; 6 Institute of Tropical Medicine, Antwerp, Belgium

**Keywords:** Social health protection, Poverty, Financial risk protection, Inequity, Universal health coverage, Hardship financing

## Abstract

**Background:**

Financial risk protection is a core dimension of universal health coverage. Hardship financing, defined as borrowing and selling land or assets to pay for healthcare, is a measure of last recourse. Increasing indebtedness and high interest rates, particularly among unregulated money lenders, can lead to a vicious cycle of poverty and exacerbate inequity.

**Methods:**

To inform efforts to improve Cambodia’s social health protection system we analyze 2019–2020 Cambodia Socio-economic Survey data to assess hardship financing, illness and injury related productivity loss, and estimate related economic impacts. We apply two-stage Instrumental Variable multiple regression to address endogeneity relating to net income. In addition, we calculate a direct economic measure to facilitate the regular monitoring and reporting on the devastating burden of excessive out-of-pocket expenditure for policy makers.

**Results:**

More than 98,500 households or 2.7% of the total population resorted to hardship financing over the past year. Factors significantly increasing risk are higher out-of-pocket healthcare expenditures, illness or injury related productivity loss, and spending of savings. The economic burden from annual lost productivity from illness or injury amounts to US$ 459.9 million or 1.7% of GDP. The estimated household economic cost related to hardship financing is US$ 250.8 million or 0.9% of GDP.

**Conclusions:**

Such losses can be mitigated with policy measures such as linking a catastrophic health coverage mechanism to the Health Equity Funds, capping interest rates on health-related loans, and using loan guarantees to incentivize microfinance institutions and banks to refinance health-related, high-interest loans from money lenders. These measures could strengthen social health protection by enhancing financial risk protection, mitigating vulnerability to the devastating economic effects of health shocks, and reducing inequities.

**Supplementary Information:**

The online version contains supplementary material available at 10.1186/s12939-023-02016-z.

## Introduction

### Background

**Fig. 1:**
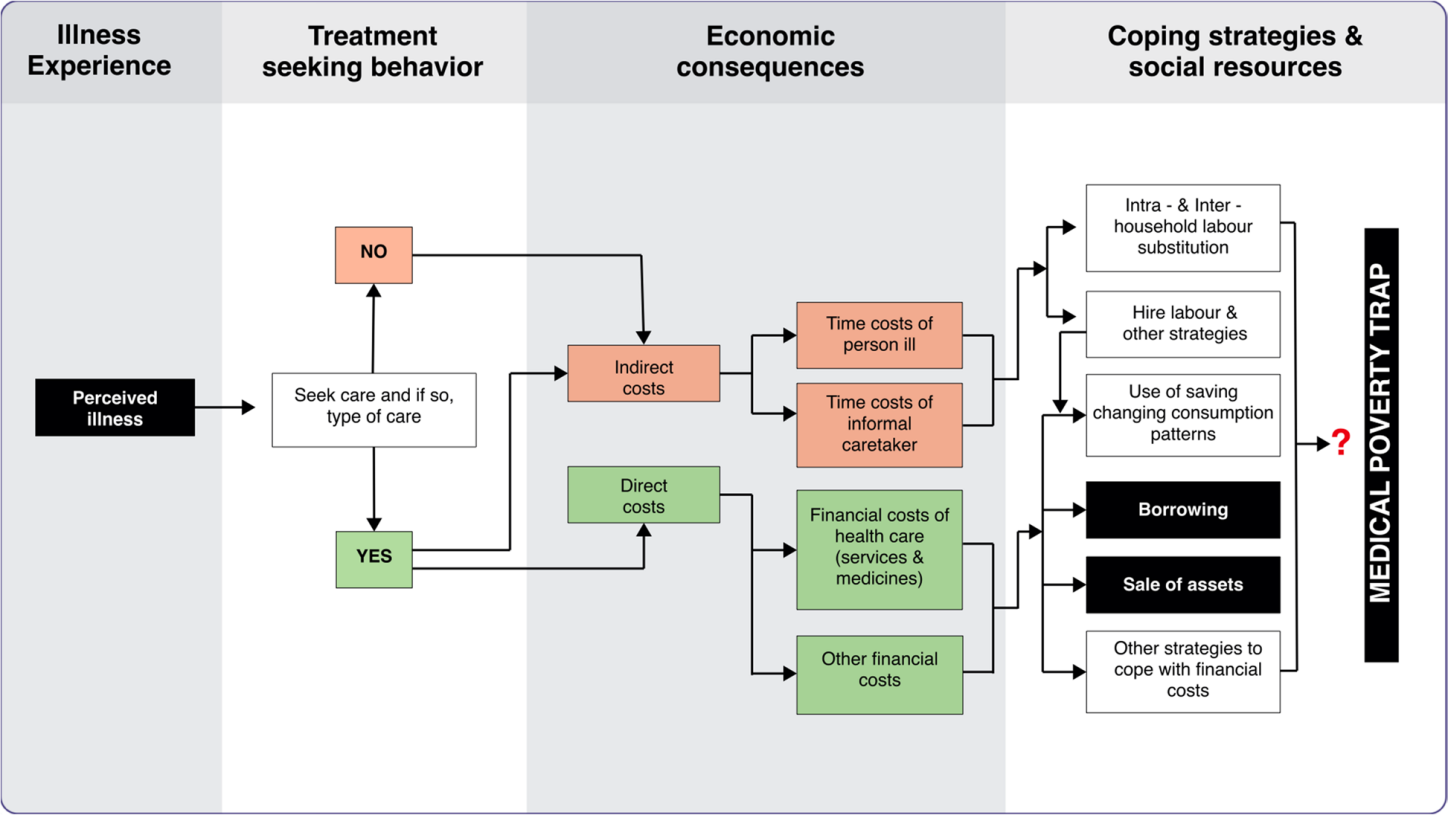
Simplified flow-chart of key issues relating to the economic consequences of illness (Revised from Social Science & Medicine, 62, McIntyre D, Thiede M, Dahlgren G, Whitehead M. *What are the economic consequences for households of illness and of paying for health care in low- and middle-income country contexts?*, p.860, Copyright (2006), with permission from Elsevier)

Universal health coverage (UHC) aims to ensure access to needed, quality health services without exposure to financial hardship. Substantial out-of-pocket medical expenditures (OOPE) can increase household economic vulnerability and lead to or exasperate poverty, particularly when ill-health leads to a loss of income [42, 45]. Health shocks can cause households to turn to hardship financing, defined as borrowing and selling assets to pay for healthcare, as a last resort [2, 13, 36]. This can undermine livelihoods and lead to a vicious circle of long-term impoverishment, inequity, health poverty, vulnerability, over-indebtedness, negative economic impacts, and low social cohesion [9, 22, 42, 62]. In addition, household debt is an important determinant of health outcomes [10]. Figure 1 illustrates the key issues relating to the economic consequences of illness.

There is also strong evidence of the positive effect of health on productivity, economic development, and poverty reduction [23, 49, 52, 61]. Arora found that changes in measures of long-term health increased the pace of national economic growth by 30–40% [4]. There are several indicators to measure financial risk and the extent of health-related expenditures (see Table 1). Flores, Krishnakumar, O’Donnell and Van Doorslaer state that “measures that ignore coping strategies not only overstate the risk to current consumption and exaggerate the scale of catastrophic payments but also overlook the long-run burden of health payments” [17]. By contrast, metrics of economic productivity loss related to illness and injury are scarce, particularly for low- and middle-income countries (LMIC). Simple and direct economic measures are needed to facilitate the regular monitoring and reporting on the devastating burden of excessive OOPE to policy makers. This is essential to ensure a continuous focus on the mitigation of hardship financing as well as to rationalize new investments in health care [2, 35].

**Table 1:** Key financial risk protection metrics and definitions

Terminology	Definition	Reference
Hardship Financing	Borrowing and selling assets, particularly land, to pay for healthcare	[36]
Distress Financing	Borrowing with interest to pay for healthcare	[24]
Capacity-to-pay	Total household consumption net of subsistence requirements, adjusted for equivalent household size	[48]
Catastrophic Expenditure	Out-of-pocket health expenditures exceeding a pre-specified percentage of consumption or income; common thresholds found in the literature are 10%, 25%, and 40%	[59]
Debt burden	Ratio of debt service to income or consumption expenditure	[47]
Over-indebtedness	Ratio of debt service to household income, exceeding a prespecified threshold	[40] [8]
Impoverishment	Extent to which people are made poor, or more poor, by spending on health	[60]

To inform efforts to improve Cambodia’s social health protection system, this study identifies risk factors associated with hardship financing and assesses the impact of hardship financing on household consumption expenditure. In addition, we estimate the annual economic productivity loss and economic burden of hardship burden as well as provide policy recommendations to mitigate the situation.

### Cambodian context

Cambodia is a lower-middle income country with a population of about 16.5 million and Gross Domestic Product (GDP) per capita at US$ 1,643 in 2019. Current health expenditure as a proportion of GDP is 6%; OOPE constitutes 57.5% of total health expenditure [63]. High OOPE is associated with catastrophic health expenditure which can impoverish households or deepen existing poverty [29, 42].

Cambodia’s overall policy of financial sector self-regulation,^1^ similar to other LMICs, has enabled the micro-finance industry to pursue an aggressive market expansion [1, 5, 21]. Cambodia is considered a microfinance‐saturated country with high levels of household debt raising concerns about over‐indebtedness [8]. In response, the government instituted an 18% cap on annual microfinance interest rates in 2017. However, to maintain profits Microfinance Institutions (MFIs) increased loan fees and loan sizes while imposing harsher penalties for late repayment [8, 21]. The Microfinance Information Exchange (MIX) Market reports a substantial increase in micro-credit borrowing in Cambodia over the past decade. Total outstanding loans increased from US$ 1.17 billion among 1.25 million borrowers in 2010 to over US$10 billion among 2.22 million borrowers in 2018 (see Appendix 1).

A recent Microfinance Index of Market Outreach and Saturation (MIMOSA) report estimates Cambodia’s credit penetration rate between 21.8 and 34.9 borrowers per 100 adults, yielding the highest saturation in the MIMOSA framework. The household debt burden is further compounded by the large and continuing growth in loan sizes [43]. In 2017, the International Monetary Fund (IMF) raised concerns about rapid credit growth in Cambodia and noted it to be the main domestic risk [8].

The issue of rapidly increasing indebtedness is compounded by the high cost of borrowing, particularly among the poor who have less collateral, and therefore limited access to loans from formal commercial banks or micro-finance institutions. Poor people often turn to informal loan providers. The use of unregulated money lenders who charge high interest rates is well documented [25, 31]. Ir et al*.* describe multiple types of informal credit for health including small, short-term loans which are typically granted for periods of 10–20 days, generally under US$ 100 with interest amounting to 20% of the loan. Longer-term health loans, commonly without a specified repayment period, can accrue a daily interest rate of 1% or a monthly rate of 5% to 30% which often results in the total interest exceeding the amount borrowed [25]. Van Damme et al*.* found interest rates among households with outstanding debt to be between 2.5–15% per month. This can lead to a vicious debt cycle of impoverishment and insolvency as productive assets including land are sold or confiscated to settle the debt [6, 31, 57]. Over-indebtedness can even force families to abandon their residence [21]. The Cambodian Children’s Fund reports that over 80% of families who relocate to the Steung Meanchey garbage dump site carry significant debt with interest rates between 10–20% per month; nearly two-thirds of the indebted families borrowed for medical treatment.

The 2014 Cambodia Demographic and Health Survey found that 20% of people reporting to be ill or injured in the past 30 days resorted to hardship financing: relying either on loans (12.4%) or on selling assets (7.6%) to pay for transport and healthcare [50]. However, national incidence of catastrophic health expenditure (i.e. OOPE exceeding 40% of capacity-to-pay) decreased from 7.1% in 2004 to 5.2% in 2009 to 4.9% by 2014 [14]. However, this positive, but slowing evolution disproportionately benefited urban households (7.3% in 2004 to 3.6% in 2014) over their rural counterparts (from 9.6% in 2004 to 7.2% in 2014) [27]. Jithitikulchai found that catastrophic health expenditure significantly decreased among poor households with sick members from 11% in 2014 to 7% in 2017 [29].

### Social health protection

The Cambodian government’s highest-level strategy and policy documents envisage the strengthening of social health protection with the reduction of poverty, vulnerability, and inequality as explicit policy goals [11]. The Rectangular Strategy Phase IV 2018 calls for a “push for UHC in Cambodia by expanding coverage of the Health Equity Fund”; the National Social Protection Policy Framework 2016–2025 aims to “…develop and expand social health protection schemes to achieve UHC”; and, the National Strategic Development Plan 2019–2023 targets “65% of the population [to be] covered by social health protection systems by 2023”.

Cambodia’s largest social health protection scheme, the Health Equity Fund (HEF), aims to provide financial risk protection to the poorest by enabling access to free public health care with the issuance of an Equity card (covering about 16% of the population). This scheme is complemented by the National Social Security Fund (NSSF) which provides social health insurance to registered private sector workers, civil servants, and some selected populations [33]. By the end of 2020, these schemes collectively covered approximately 30% of the population [34]. They serve as the foundational elements for Cambodia to achieve financial risk protection and universal health coverage.

However, there is evidence showing that Health Equity Fund members still borrow to pay for healthcare. Jacobs et al*.* found that 83% of payment-exempted patients resorted to borrowing, on average, 3.4 times the total direct costs of the illness episode; by comparison 48% of paying patients borrowed, at a rate of 0.74 (i.e. less than) the total direct costs [28]. In a separate study, 82% of payment-exempted patients borrowed 6.6 times the total direct costs relating to the illness. It is hypothesized that the excessive borrowing (in relation to direct costs) by patients entitled to free care is due to opportunity costs related to the illness [25]. Another study found that the Health Equity Fund did not reduce the likelihood of incurring health-related debt, but did reduce the amount of that debt [16].

### Productivity and economic impact

Increased productivity can contribute to the prevention and reduction of vulnerability, poverty and inequality, ultimately leading to increased human capacity and economic growth [51]. Improving labor productivity is fundamental for Cambodia to remain competitive, particularly given rising competition from other low-wage garment exporting countries [64]. Estimating the economic cost of illness and injury requires the quantification of productivity loss as well as the appropriate assignment of a monetary value to that loss. The former can be estimated by recording the number of days a person stopped doing their usual activities. There are three primary methods for estimating the latter: (1) salary conversion; (2) introspective methods; and, (3) estimating the cost of countermeasures related to absenteeism (i.e. absence from work) and presenteeism (i.e. reduced productivity while at work) [41]. A fourth “human capital approach”, similar to salary conversion, uses prevailing wage rates. These methods typically take the employer perspective, assigning a lower value to conditions that are more frequent among low wage earners or those not in the workplace, do not account for lost future earnings, and underestimate the true cost of illness and injury [15]. Furthermore, most cost-of-illness studies are done in high-income countries and focus on a specific condition or illness [44]. As such, these methods are problematic for contexts with high informal sector populations such as in most low- and middle-income countries (LMIC). The dearth of studies on the economic costs of illness in LMIC exemplifies the need for a more generalized approach that can be conducted routinely and measures the productivity loss and economic impact associated with illness and injury, particularly in countries with high levels of informal workers.

## Methods

### Data

This study analyzes the nationally representative household data from the 2019–2020 Cambodia Socio-economic Survey completed by the National Institute of Statistics, Ministry of Planning. The dataset contains information on demographic characteristics, housing, education, labor force, household income, consumption, liabilities, and healthcare for 10,075 households, including 5,614 households with at least one member having reported an illness or injury in the past 30 days. Both land and asset selling for health are included when reporting hardship financing in the past 30 days as this was recorded for households indicating an illness or injury. However, report of hardship financing for health in the past year excludes non-land asset selling as it was not included as a survey response option. Thus, we calculate monthly hardship financing by aggregating households reporting any health care seeking in the past 30 days that was financed by borrowing or selling assets. We calculate annual hardship financing by aggregating all households reporting the sale of land to address family health issues and loans taken for illness, injury, or accident. Items included in consumption expenditure are listed in Appendix 2.

### Statistical methods

First, this study describes the characteristics of unproductive household debt, defined as debt taken for purposes that are not directly associated with revenue generation. We calculate descriptive statistics for the period of the debt, time to full repayment, source of the loan, primary purpose of the loan, total amount borrowed, current outstanding debt, monthly interest rate, and estimate the total outstanding healthcare debt.

Second, we use two-stage Instrumental Variable (IV) Probit regression to identify factors that explain the risk of hardship financing at the household level. Independent variables used in the model are: (1) log OOPE (continuous); (2) total non-productive days in the household due to illness or injury in the past 30 days (discrete); (3) savings used to finance healthcare (binary); and, (4) log net income (continuous). Net income is an endogenous variable as report of household income in a survey is well-known to contain measurement error related to under-reporting. In addition, income is likely correlated with unobserved factors that are also directly correlated with the dependent variable hardship financing, most notably mortality. Finally, there is also potential for reverse causality as hardship financing may affect net income, particularly related to debt and interest repayment. To address endogeneity net income is instrumented by head of household age and total adult years of education in the household; this restricts the effect of net income on the error term [3, 32]. The two primary conditions to use these IVs are: (1) age and education must have a casual effect on net income, and (2) age and education do not have a direct influence on consumption expenditure. We find strong empirical evidence that the first condition is met; diagnostic test statistics relating to endogeneity, under identification, over identification, and weak identification are presented in Appendix 3. The second condition cannot be directly tested because the error is inherently unobservable. We are unable to identify any evidence or theoretical rationale in the literature that would explain how age of the head of household or education would deterministically impact on hardship financing other than through income. Equation 1 expresses the final hardship financing risk model.

1 $$\begin{array}{c}{Hardship\_Financing}_{i}={\mathrm{B}}_{0}+{\mathrm{B}}_{1} \mathrm{ln}\_{\mathrm{OOPE}}_{\mathrm{i}}+\\ {\mathrm{B}}_{2}\mathrm{Total}\_\mathrm{non}\_\mathrm{productive}\_{\mathrm{days}}_{\mathrm{i}}+{\mathrm{B}}_{3}\mathrm{Savings}\_{\mathrm{spent}}_{\mathrm{i}}+\\ {\mathrm{B}}_{4}\mathrm{ln}\_\mathrm{net}\_\mathrm{income}\;{ \left[\mathrm{IVs}:\mathrm{Age}\_\mathrm{household}\_\mathrm{head},\mathrm{ Adult}\_\mathrm{years}\_\mathrm{edu}\right]}_{1}+\upvarepsilon \end{array}$$

The analysis tested other factors that could explain hardship financing including Covid-19 period,^2^ household size, educational level of head of household, age of head of household, age of head of household squared, sex of head of household, provider type (i.e. public, private, non-medical, and overseas), hospitalization (i.e. yes or no), number of inpatient days, chronic disease, number of people over sixty years of age in the household (discrete), disabled people in the household (binary), and total working age adults (i.e. 15–59 years) in the household. The model initially included Equity card as an independent variable; however, as the issuance of an Equity card is means-tested, it is potentially collinear with net income and therefore excluded. Other variables tested for model fit were any member of the household reporting to have an NSSF social health insurance card (i.e. yes or no), Phnom Penh residence (i.e. yes or no), and any current loan with an MFI or bank (i.e. yes or no). Descriptive statistics for all variables of interest are presented in Table 2. These variables were excluded from the final hardship financing model as they did not improve the fit as evaluated using the log ratio test [12]. To limit the influence of outliers, net income and consumption expenditure data were winsorized to transform values below the 1^st^ percentile to the 1^st^ percentile and values above the 99^th^ percentile to the 99^th^ percentile [20]. Analyses were adjusted for sample design; Stata 17 was used for data management and analysis [56].

**Table 2:** Key variable summary statistics and expected direction in relation to dependent variables by model

	Units	Mean	Median	Std. Dev	min	max	Expected Direction *Model 1*	Expected Direction *Model 2*
*Hardship Financing Model 1*								
Hardship financing (past 30 days)^a^	binary	.03	0	.17	0	1	n.a	n.a
*Consumption Expenditure Model 2*								
Monthly non-medical expenditure^a^	US$	388.81	324.38	248.21	75.89	1530.91	n.a	n.a
Monthly non-food, non-medical exp.^a^	US$	148.81	96.22	160.07	9.86	1013.84	n.a	n.a
Monthly food expenditure^a^	US$	238.49	213.05	123.26	58.19	719.06	n.a	n.a
*Variables of interest*								
Monthly net income^b^	US$	1837.88	597.56	4188.99	-1888.35	28,081.40	**-**	** + **
Hardship financing (past 12 months)	binary	.02	0	.14	0	1	n.a	**-**
Equity card	percent	.10	0	.3	0	1	**-**	**-**
NSSF card^c^	percent	.14	0	.35	0	1	**-**	n.a
Out-of-pocket health expenditures	US$	37.68	3.66	183.07	0	13,853.66	** + **	**-**
Non-productive days	days	.86	0	4.32	0	65	** + **	**-**
Savings spent for healthcare	percent	.01	0	.10	0	1	** + **	**-**
Head of household age^c^	years	48.32	48	13.84	17	96	n.a	n.a
Head of household sex (female)	percent	.20	0	.40	0	1	** + **	**-**
Head of household education	years	8.27	6	12.62	0	88	**-**	** + **
Cumulative total adult education^c^	years	17.9	14	14.9	0	122	n.a	** + **
Household size	people	4.42	4	1.77	1	17	**-**	** + **
Adults over 60 years	people	.43	0	.69	0	4	** + **	** + **
Disabled household member	percent	.10	0	.29	0	1	** + **	** + **
Adults of working age	people	2.68	2	1.42	0	12	**-**	** + **
Care-seeking at public provider	percent	.22	0	.41	0	1	**-**	** + **
Hospitalization in past 30 days	percent	.06	0	.25	0	1	** + **	**-**
Cumulative in-patient days	days	.28	0	1.71	0	52	** + **	**-**
Report of current illness for > 1 year	percent	.46	0	.50	0	1	** + **	**-**
Chronic illness type reported	percent	.07	0	.26	0	1	** + **	**-**
Covid-19 period	percent	.38	0	.48	0	1	** + **	**-**
Residing in Phnom Penh	percent	.09	0	.29	0	1	**-**	** + **
Current MFI or bank loan	percent	.31	0	.46	0	1	**-**	** + **

Table statistics are not weighted

*n.a.* not applicable for collinear, dependent and instrumental variables

^a^Denotes dependent variables

^b^Denotes instrumented variable

^c^Denotes instruments

Third, testing all variables described above, we fit a multivariate two-stage least squares instrumental variable (2SLS IV) regression model to assess the impact of hardship financing on household non-medical consumption expenditure, food consumption expenditure, and non-food/non-healthcare consumption expenditure. All consumption expenditure data was transformed to a one-month period. There is risk of bias when assessing the impacts of health shocks on household economic outcomes [2]. This is particularly problematic as it is important to control for income which is highly correlated with consumption expenditure. In addition to the endogeneity issues noted above, there are additional unobserved factors that are likely correlated with both income and consumption expenditure such as savings, negative personal circumstances, food prices, food preferences, social networks, and thriftiness [7, 18, 30]. Finally, household income is likely to shift as part of a functional relationship between other variables within the model, specifically the primary variable of interest: hardship financing. For example, a loan or sale of land can be expected to (temporarily) increase household income which could lead to an increase in consumption expenditure.

To address this endogeneity, we employ 2SLS IV regression. This restricts the correlation of household income with the error term, thus limiting the effect to operate only through modeled household net income and the other independent variables. The instrumental variables used for this equation are: (1) the age of the head of household (discrete); and (2) NSSF card (binary, indicating formal, registered employment of at least one member of the household). As noted above, there are two primary conditions to use these IVs. For this model, the first condition requires that age and formal employment have a casual effect on net income; the second condition necessitates that age and formal do not have a direct influence on consumption expenditure. We find strong empirical evidence that the first condition is met; diagnostic test statistics relating to endogeneity, under identification, over identification, and weak identification are presented in Appendix 4. As noted above, the second condition cannot be directly tested because the error is inherently unobservable. We are unable to identify any evidence or theoretical rationale in the literature for how age of the head of household or formal employment would deterministically impact on consumption expenditure other than through their effect on net income. The model controls for other explanatory factors as shown in Eq. 2.

2 $$\begin{array}{c}{Consumption\_Expenditure}_{i}={\mathrm{B}}_{0}+{\mathrm{B}}_{1}{\mathrm{Hardship}\_\mathrm{Financing}}_{\mathrm{i}}+{\mathrm{B}}_{2}{\mathrm{HHsize}}_{\mathrm{i}}+\\ {B}_{3}{\mathrm{Female}\_\mathrm{head}}_{i}+{\mathrm{B}}_{4}{\mathrm{PhnomPenh}\_\mathrm{residence}}_{\mathrm{i}}+{\mathrm{B}}_{5}{\mathrm{MFI}\_\mathrm{Bank}\_\mathrm{loan}}_{\mathrm{i}}+{\mathrm{B}}_{6}{\mathrm{Covid}-19}_{\mathrm{i}}+\\ \begin{array}{c}{\mathrm{B}}_{7}{\mathrm{Working}\_\mathrm{age}\_\mathrm{HHmembers}}_{\mathrm{i}}+{\mathrm{B}}_{8}{\mathrm{Working}\_\mathrm{age}\_\mathrm{HHmembers}}_{\mathrm{i}}^{2}+\\ {\mathrm{B}}_{9}{\mathrm{ln}\_\mathrm{net}\_\mathrm{income}}_{\mathrm{i}}\left[\mathrm{IV}:\mathrm{ Age}\_\mathrm{household}\_\mathrm{head},\mathrm{ NSSF}\_\mathrm{card}\right]+\upvarepsilon \end{array}\end{array}$$

We calculate the economic impact of lost productively due to illness or injury using Eq. 3.

3 $$\left(\frac{GDP}{\left(working\;age\;pop. * working\;days\;per\;year\right)-Nonproductive\;days} \right)*Nonproductive\;days=GDP\; loss$$

First, we estimate the value for one (1) workday by dividing 2019 GDP by the total number of workdays. The latter is calculated by multiplying the 2019 working age population by the number of working days in 2019 net the estimated total non-productive days due to illness or injury. The value for one (1) day of work is then multiplied by the estimated total non-productive days due to illness: this represents the lost GDP. Finally, the proportion of GDP lost is calculated by dividing the estimated GDP lost by the sum of GDP and GDP lost.

The annual economic burden related to hardship financing is estimated by summing the total health-related loan principal and interest with health-related land sale income over the past year. We consider land sale primarily for health purposes as an expense similar to spending savings. This is because sale of land is a loss of a productive asset, and the proceeds of the sale are reported for non-productive purposes (i.e. paying for health related issues). This approach is further rationalized in the discussion section.

Finally, we estimate the total hardship burden and the potential interest savings among health-loan borrowers for three annual interest rate cap scenarios (18%, 12%, and 8%). First, the total interest is calculated by multiplying the principle, monthly interest rate, total repayment period in months. Second, we adjust the monthly interest rate for all health-loans over each interest rate scenario to the cap and calculate the modeled interest. The difference between the total interest and modeled interest represents the potential interest rate savings.

## Results

Overall, 2.7% [95%CI: 2.4–3.1%] of households, estimated to represent 98,505 households nationally, report hardship financing to pay for healthcare in the past 12 months: 1.3% [CI: 1.0–1.5%] report distress financing for health (i.e. borrowing money with interest); 1.3% [CI: 1.1–1.6%] report selling land; and, 0.05% [CI: 0.02–0.11%] report both borrowing and selling land.

### Borrowing

Over one-third (36.5%) [CI: 35.1–38.0%] of households report having at least one loan of any type. Nearly 70% [CI: 68.3–70.9%] of households with loans are over-indebted with their debt payment exceeding 50% of their total consumption expenditure. About 28.3% [CI: 26.8–29.9%] of all households hold unproductive loans. Thus, unproductive loans account for over two-thirds (68.7%) [CI: 66.5—70.8%] of all loans. The primary uses of unproductive loans are for household consumption (37.5%), purchase or improvement of the dwelling (25.6%), purchase of consumable durables (21.4%), service existing debts (8.1%), illness, injury or accident (5.5%), and (1.9%) for rituals such as weddings and funerals.

**Fig. 2:**
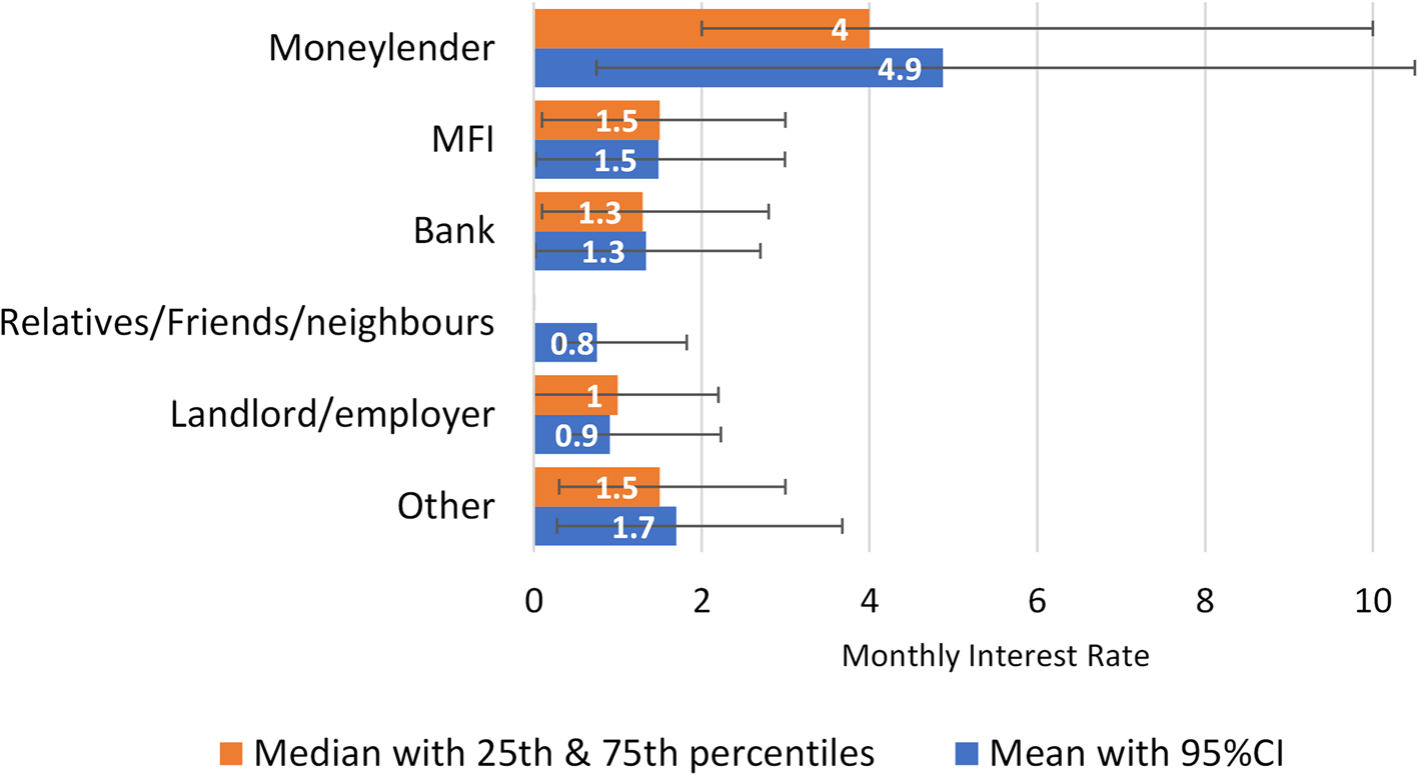
Median and mean monthly interest rates by lender type

The median period of unproductive debt is 36 months with the median period to full repayment 23 months. In relation to health loans, the median debt period is 27 months; the median period to full repayment is 17 months. The median loan size for health is US$ 975.60 with a median monthly repayment amount of US$ 50.73 inclusive of principal and interest. At the population level, we estimate 50,122 households hold a total amount of outstanding health debt in the amount of US$ 88.2 million [CI: US$ 51.9–124.0 million]. Moneylenders charge 2.5–3.4% higher monthly interest rates compared with Microfinance Institutions, and 2.7–3.6% higher compared with banks (Fig. 2).

**Fig. 3:**
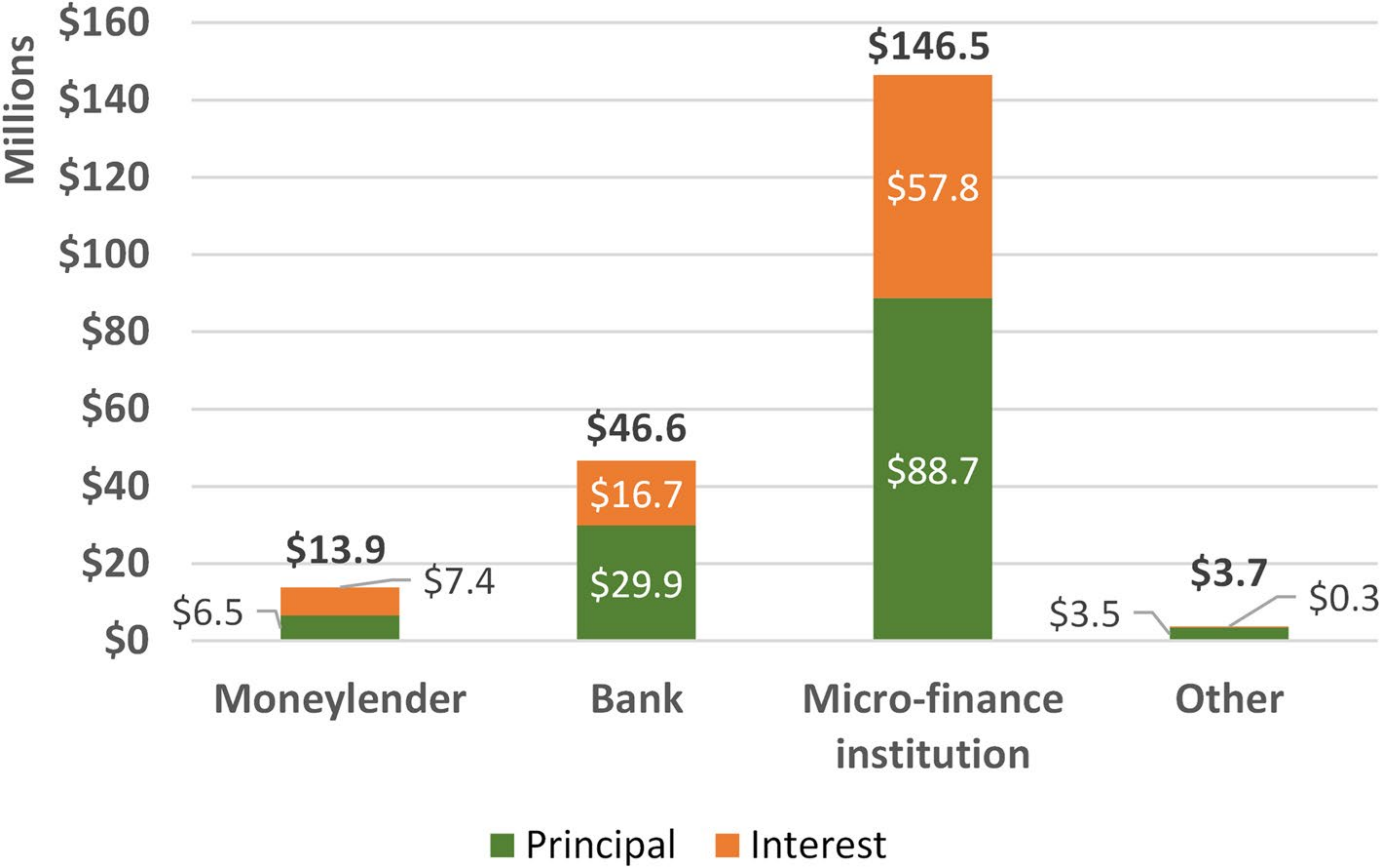
Loans for health in the past 12 months: total debt, principal and interest by major lender type

Figure 3 shows the total principal and interest burden for health-related loans taken in the past year. Moneylenders account for 3.6% of these loans (not shown). However, the total related debt (principal and interest) accounts for 6.6% or US$ 13.9 million; over half of that debt (US$ 7.4 million) is due to interest (see Appendix 2). Banks are estimated to provide 39.4% of health-related loans (not shown), however only account for 22.1% of the debt estimated at US$ 46.6 million. MFIs account for 48.1% of health-related loans (not shown), but 69.4% of the debt or US$ 146.5 million. Other lenders including family, friends, neighbors, landlords, and employers account for 8.9% of health-related loans (not shown); these loans amount to 1.8% of the total health-related debt.

In relation to over-indebtedness for health, nearly one-quarter (23.4%; CI:16.3–32.3%) of households with health-related debt report their health debt payment to exceed 25% of their consumption expenditure.

### Selling land

**Fig. 4:**
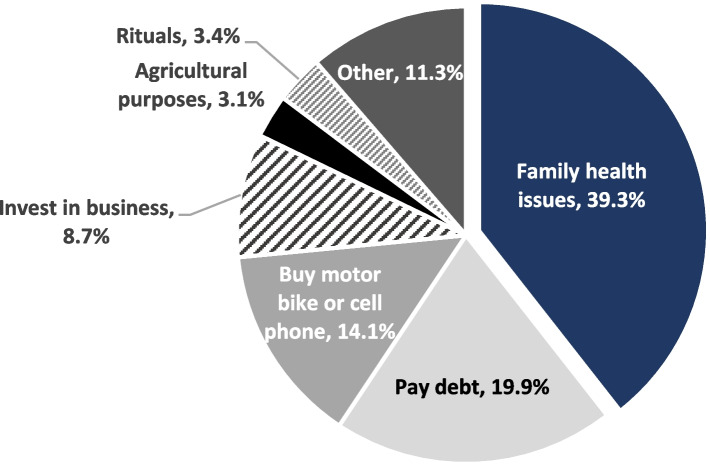
Primary reasons for selling land in the previous 12 months

Figure 4 illustrates the primary reasons for selling land in the past year. Among the 3.4% [CI: 3.0- 3.9%] of households that report doing so are for family health issues (39.3%) and paying debt (19.9%). Households also report selling land to buy a motor bike or cell phone (14.1%), invest in business (8.7%), agricultural purposes (3.1%), rituals such as weddings and funerals (3.4%), and other purposes (11.3%) (see Fig. 3).

### Determinants of hardship financing

There are several factors which explain hardship financing for health in Cambodia (see Table 3). After controlling for other covariates in the model, characteristics that significantly predict resort to hardship financing for health are increased OOPE (*p* < 0.001), non-productive days associated with the illness or injury (*p* < 0.01), and spending of savings on the illness or injury (*p* < 0.05). An increase in net income is associated with a decreased likelihood of hardship financing (*p* < 0.001).

**Table 3:** Hardship financing predictive factors

	(1)	(2)	(3)
Variables	Structural Equation	First Stage Least Squares IV regression Net Income (logged)	Second Stage Probit IV regression
OOPE (logged)	0.339***	0.020	0.022***
	(0.028)	(0.020)	(0.003)
Non-productive days	0.014**	-0.003	0.003**
	(0.005)	(0.005)	(0.001)
Savings spent	0.606**	0.112	0.092*
	(0.213)	(0.292)	(0.044)
Net income (logged)^a^	-0.043*		-0.040***
	(0.020)		(0.009)
Age of head of household^b^		0.009***	
		(0.002)	
Adult years of education^b^		0.023***	
		(0.002)	
Constant	-2.854***	5.492***	0.216***
	(0.163)	(0.146)	(0.056)
Observations	5,312	5,312	5,312
F-statistic	44.1	71.8	22.1

Standard errors in parentheses

^***^p < 0.001, **p < 0.01, *p < 0.05

^a^Denotes instrumented variable

^b^Denotes instruments

### Economic impact of hardship financing on the household

The impact of hardship financing (over the past year) on household consumption expenditure was assessed using multivariate 2SLS IV regression. Table 4 presents the results from the structural equation (column 1), the first stage regression (column 2), and the second-stage results for total non-medical consumption expenditure (column 3), non-food, non-medical consumption expenditure (column 4), and food consumption expenditure (column 5). The following narrative focuses on the results presented in columns 3–5.

**Table 4:** Estimated effects of hardship financing (over the past 12 months) on household consumption expenditure (over the past 30 days), controlling for other key factors

	Structural Equation: Least Squares Total non-medical Expenditure (logged)	First Stage Least Squares Net Income (logged)	Second Stage Least Squares IV Total non-medical Expenditure (logged)	Second Stage Least Squares IV non-food non-medical Expenditure (logged)	Second Stage Least Squares IV Food Expenditure (logged)
Variables	(1)	(2)	(3)	(4)	(5)
Hardship financing	-0.131***	-0.268*	-0.089*	-0.086	-0.115***
	(0.034)	(0.117)	(0.039)	(0.062)	(0.032)
Household size	0.049***	-0.027	0.052***	0.035***	0.063***
	(0.005)	(0.019)	(0.006)	(0.062)	(0.005)
Female head of household	-0.131***	0.082	-0.153***	-0.196***	-0.133***
	(0.016)	(0.049)	(0.018)	(0.028)	(0.015)
Phnom Penh residence	0.424***	0.924***	0.271***	0.167**	0.339***
	(0.027)	(0.098)	(0.043)	(0.063)	(0.038)
Any MFI or bank loan	0.232***	0.010	0.231***	0.526***	0.037**
	(0.014)	(0.055)	(0.016)	(0.026)	(0.013)
Covid-19	-0.051**	0.023	-0.062*	-0.011	-0.091***
	(0.020)	(0.086)	(0.025)	(0.026)	(0.020)
Working-age household members	0.179***	0.094	0.171***	0.291***	0.126***
	(0.015)	(0.049)	(0.017)	(0.027)	(0.014)
Working-age household members (squared)	-0.018***	0.000	-0.019***	-0.032***	-0.014***
	(0.002)	(0.007)	(0.002)	(0.004)	(0.002)
Net income (logged)^a^	0.029***		0.192***	0.322***	0.112***
	(0.005)		(0.034)	(0.052)	(0.032)
Age of health of household^b^		0.008***			
		(0.002)			
NSSF card^b^		0.540***			
		(0.064)			
Constant	5.001***	5.708***	4.012***	1.778***	4.171***
	(0.041)	(0.139)	(0.212)	(0.326)	(0.178)
Observations	9,447	9,447	9,447	9,447	9,447
F-statistic	161.2	41.24	116.92	103.42	111.97
R-squared	0.23	-	-0.041	-0.075	0.104

Standard errors in parentheses

^***^ *p* < 0.001, ** *p* < 0.01, * *p* < 0.05

^a^Denotes instrumented variable

^b^Denotes instruments

Hardship financing is associated with an overall 8.9% decrease in total non-medical consumption expenditure (*p* < 0.05), and a decrease of 11.5% in food expenditure (*p* < 0.001) after controlling for other significant covariates, including net income. In addition, non-medical consumption expenditure increases by 5.2% with each additional household member (*p* < 0.001). Female headed household’s non-medical expenditure is 15.3% (*p* < 0.001) less compared to male headed households. Non-medical expenditure is 27.1% (*p* < 0.001) higher for households in Phnom Penh compared to the rest of the country, this is mostly attributable to higher food expenditure (33.9%, *p* < 0.001). Households reporting any type of micro-finance or bank loan have higher non-medical expenditure (23.1%, *p* < 0.001), with non-medical, non-food expenditure 52.6% higher (*p* < 0.001) and food expenditure 3.7% higher (*p* < 0.01). Non-medical spending was 6.2% (*p* < 0.01) lower during the Covid-19 period; this is driven by a 9.1% decrease in food expenditure (*p* < 0.001).

Household labor supply is an important determinant of expenditure as non-medical expenditure increases by 17.1% (*p* < 0.001) for every working-age adult in the household. However, the association is non-linear as the quadratic term (i.e. working-age household members squared) indicates that the effect of increased labor supply eventually decreases non-medical consumption expenditure (*p* < 0.001) likely attributable to economies of scale within the household. Finally, a 1% increase in net household income (instrumented by age of head of household and NSSF card) increases non-medical expenditure by 19.2% (*p* < 0.001), non-food, non-medical expenditure increasing by 32.2% (*p* < 0.001) and food expenditure by 11.2% (*p* < 0.001).

### Productivity loss and economic cost

As discussed above, productivity loss is a significant determinant of hardship financing. Among households reporting any illness or injury in the past 30 days, the mean number of lost productive days (i.e. when the individual stopped doing usual activities) is 1.54 [CI: 1.40–1.70]. Among households reporting non-productive days in the past 30 days, the mean number of days lost is 11.95 [CI: 11.1–12.8].

Using Eq. 3, we estimate the GDP contribution of one person-day at US$ 12.01. This yields an estimated total annual lost productivity due to illness or injury of US$ 459.9 million with uncertainty limits (UL) of US$ 395.1—US$ 524.6 million. This represents an annual loss of GDP of 1.7% [UL 1.4–1.9%] in 2019.

Table 5 presents population-level estimates and corresponding 95% confidence intervals relating to the household economic cost of health-related hardship financing. Over the past 12 months the principal on health loans amounts to US$ 129 million; these loans carry a total interest burden of US$ 82.1 million. Total lost wealth due to land sale in the past 12 months amounts to US$ 39.7 million. Thus, the total annual household economic cost due to hardship financing is estimated at US$ 250.8 million [CI: US$ 154.8 – 346.4 million] and equates to 0.9% of GDP [CI: 0.6–1.3%] in 2019.

**Table 5:** Economic cost from illness and injury among households: health-related loans, interest, and sale of land for health purposes in US$

Hardship Burden Category	Point Estimate	Std. Err	95%	CI
Health loan principal	129,000,000	22,700,000	83,800,000	174,000,000
Health loan interest	82,100,000	14,700,000	52,900,000	111,000,000
Health loan burden	211,100,000	22,900,000	136,700,000	285,000,000
Land sale income for health	39,700,000	10,900,000	18,100,000	61,400,000
Total hardship burden	250,800,000		154,800,000	346,400,000
Proportion of GDP	0.9%		0.6%	1.3%

Finally, we estimate the potential reduced economic burden of health debt using three refinancing scenarios: capping monthly interest on all health debt at 1.5%, 1.3%, and 1%; calculating this over the loan period would reduce the total interest burden by US$ 4.8 million, 10.1 million, and 21.2 million, respectively.

## Discussion

### Limitations

The Cambodia Socio-economic Survey was not designed specifically to investigate hardship financing. Thus, the analysis was limited in several aspects. Specifically, this prohibited a more comprehensive examination of the characteristics of health shocks and hardship financing. For example, the hardship financing regression model (1) was limited to report of hardship financing within the past month as health care-seeking indicators were limited to that period. This is because hardship financing over the past year cannot be plausibly explained by health and related care-seeking in the past 30 days. Thus, we were unable to link the amount of the financial burden due to sale of assets and debt related to health issues directly with the illness or injury episode as the former was reported for the past 12 months.

In addition, there are many risks for bias when analyzing health shocks. Two-way causality or endogeneity between economic outcomes and health events and unobserved characteristics of the household may increase illness susceptibility and economic severity can lead to bias [2]. Reduced consumption expenditure, particularly food consumption expenditure, has the potential to increase vulnerability to adverse health events over time. To address this constraint, we limited the effect of bias relating to net income by using instrumental variables. In relation to the consumption expenditure Eq. (2), it is important to note that the food consumption expenditure data is based on one-week recall, and the primary explanatory variable of interest, hardship financing, is reported over the past year. This reduces the plausibility of reverse causality. As noted above, reverse causality is a potential issue as a health shock could be caused by or result in a chronic health condition. However, we tested two chronic disease variables (i.e. report of illness in the past 30 days coming and going for the past year; and, report of illness classified as chronic such as high blood pressure or diabetes). These variables were excluded from the final model as neither was found to be significant. Furthermore, borrowing or asset selling could cause a temporary increase in consumption expenditure. This could be expected to reduce the likelihood of finding statistically significant negative impacts on consumption expenditure, which suggests that the estimates presented in this study may be conservative.

The survey asked how many days an ill or injured individual in the household stopped doing usual activities. Although the calculation of productivity loss could be restricted to adults of working age, we believe it is important to count all reported days lost in the household. First, it is reasonable to assume that children and older persons who stop doing their usual activities require an adult member of the household to also stop doing their usual activities to provide care. Second, the survey does not capture presenteeism, or a level of reduced productivity due to illness or injury. Therefore, we consider that limiting productivity loss to the reported activity stoppage yields a conservative estimate.

Further to the description in the methods section, the annual economic cost of hardship financing is estimated by summing the total health-related loan principal and interest with health-related land sale income over the past year. This method assumes that the land sale value is of the same magnitude of the direct and in-direct health-related costs. Although it can be argued that this may not be the case, we believe that it can be considered a good or even conservative proxy for several reasons. First, in Cambodia land can be sub-divided into relatively small units which provides the seller the possibility to only sell what is considered necessary vis-à-vis the primary purpose. Second, selling land under distress such as in a health crisis would likely give the purchaser negotiating leverage, thus minimizing any incentive for the seller to “over sell”. Third, the method does not account for future loss of income related to the sale. Fourth, it does not incorporate sale of non-land assets. Lastly, it does not capture households with no opportunity to borrow or land to sell rendering it impossible to seek care following a health shock [53]. Moreover, we believe it is important to estimate the economic/monetary cost for hardship financing as it is easier to communicate to policy makers. Finally, the estimate can be made using regularly available survey data, so it is easy to replicate and therefore monitor over time.

### Interpretation

Hardship financing is a measure of last resort for households facing health shocks. The measure explicitly captures inability to pay as well as indirect and opportunity costs [36]. Direct costs can be relatively minor compared to the large indirect cost burden from illness [54].

Due to potential collinearity, we did not assess the Equity card as a hardship financing explanatory factor. As an Equity card entitles all members of the household to free public healthcare financed under the Health Equity Funds, one would hope to find possession of the card to provide financial risk protection. However, further to the evidence presented in the introduction, Ir et al. found a significantly higher proportion of households holding an Equity card (24.7%) resorted to borrowing with interest to pay for healthcare compared to non-entitled households (12.5%) [24]. Another study found that Equity card households benefit most when health care-related costs are low, however it fails to provide the same degree of financial protection when costs are high or accrue over time, even among beneficiaries seeking care from public facilities [29].

This study found both OOPE and productivity loss to be factors that significantly increase the likelihood of hardship financing. This underscores the importance that the economic cost of illness and injury is not limited to the direct cost of healthcare or OOPE. In traditional economic analyses OOPE is cited as evidence of willingness to pay and viewed as a potential funding source to be pooled through social health insurance mechanisms. However, hardship financing underscores that paying for healthcare does not equate to ability to pay [62]. We did not find households reporting at least one member with an NSSF card significantly associated with hardship financing. The NSSF card currently only provides coverage for the formally employed worker, not the household. Thus, its potential protective affect is diluted.

Spending of savings to pay for healthcare is also significantly associated with hardship financing as this strategy increases economic vulnerability of the household. This is consistent with other evidence that households tend to first use savings when available, then resort to credit and/or selling productive assets [13].

When assessing the household economic impact of borrowing or selling land to pay for healthcare in the past 12 months results show that hardship financing is statistically associated with a 11.5% decrease in food consumption expenditure, after controlling for other statistically significant factors including net income. This evidences the longer-term impact of hardship financing on the household’s well-being and is consistent with multiple other studies showing health shocks can lead to reduced consumption [2, 58].

Given the relatively low proportion (2.7%) of the population resorting to hardship financing, the economic impact can easily be overlooked by policy makers. However, this equates to US$ 250.8 million or 0.9% GDP. In addition, the economic burden from the annual lost productivity due to illness or injury is substantial as the total economic loss amounts to US$ 459.9 million or 1.7% of GDP. This is a conservative estimate as it does not explicitly incorporate future lost earnings due to premature mortality.

It is important to recognize that microfinance plays a role in health financing. In Cambodia most MFIs actively market, encourage and grant loans for non-productive assets and activities [8]. This study found that MFIs account for nearly half (48.1%) of health loans and 69.4% of health debt including US$ 88.7 million in principal and US$ 57.8 million in interest. We also found that households holding any MFI or bank loan to have higher non-medical consumption expenditure, food expenditure, and non-food, non-medical expenditure, after controlling for other factors including net income. Evidence from Thailand and Indonesia shows that access to credit and microfinance institutions helps smooth consumption against health shocks [19]. Likewise, a study in Bangladesh found that the household sale of livestock to pay for healthcare, presenting a significant long-term cost, can be mitigated with microcredit [26]. And, a quasi-experimental study from India found that debt was the principle mitigating mechanism when faced with a health-shock which lead to significant increases in indebtedness [46].

Ir et al*.* call for research to “investigate whether extending microcredit to the poor can be used as a means to avert borrowing from informal creditors for health care expenses, and how this might be done” [25]. Although this is beyond the scope of this study, we present related policy recommendations.

First, the institutional and legal environment can increase or reduce the risks of over-indebtedness [55]. Government has a role to play relating to social services, safety nets, and regulation that could limit credit market saturation and predatory lending [8]. This is particularly relevant for people who are vulnerable due to health shocks which force households to make difficult decisions that can undermine their economic well-being. There are several possible policy levers which can increase financial risk protection, a multi-pronged approach is recommended. As discussed above, Equity card households are at higher risk of financial hardship. This social health insurance mechanism should provide financing risk protection during serious health shocks which lead beneficiaries to borrow and sell land. A qualitative study is needed to better understand the dynamics of financial risk protection among Equity card households whose members are entitled to free public health care with a few minor exceptions (most notably cancer treatment). The program should develop a catastrophic health coverage mechanism to provide financial risk protection for serious illness and injury.

Health savings and loans products require careful design to optimize value and minimize risk [39]. MFIs and banks could refinance health-related loans with high interest rates and develop a range of low-interest health loan products. Refinancing all health-related loans to the current 18% interest cap would reduce the total interest burden by US$ 4.8 million. Given the seriousness of the issue of health debt, a lower interest cap could be set for health-related loans. For example, refinancing all health loans at an annual 12% cap, equivalent to 1% monthly, has the potential to decrease the total interest burden by US$ 21.2 million; and, decreasing the annual rate to 8% annually (or 0.67% monthly) could decrease the total interest burden by US$41.1 million. High risk borrowers with limited or no collateral using informal lenders can be initially targeted through the Health Equity Fund and transitioned to lower interest rate health loans from formal lenders by offering loan guarantees. However, these approaches would require national coverage to reduce hardship financing and catastrophic expenditure at the national level. It is also important to acknowledge that there are some inherent risks related to working with profit-oriented MFIs and introducing new loan products. These approaches should be complemented with consumer protection measures through regulation and oversight mechanisms.

Cambodia is accumulating experience directly related to these recommendations. The Cambodia Children’s Fund has successfully demonstrated the importance and feasibility of transitioning informal, health loans with high interest rates among the absolute poor. The Association of Banks in Cambodia and Cambodia Microfinance Association recently called for its members to follow the National Bank of Cambodia’s 2020 directive on credit restructuring. These measures include waiving penalties, easing terms of emergency loans, and cutting interest rates [38]. And, the Royal Government of Cambodia recently launched a $200 million credit guarantee program for small and medium sized businesses targeting the agriculture, industry and service sectors [37].

## Conclusions

More than 98,500 households or 2.7% of the total population resorted to hardship financing over the past year. Factors significantly increasing risk are higher out-of-pocket healthcare expenditures, illness or injury related productivity loss, and spending of savings. The economic burden from annual lost productivity from illness or injury amounts to US$ 459.9 million or 1.7% of GDP. The estimated household economic cost related to hardship financing is US$ 250.8 million or 0.9% of GDP. Decision makers can mitigate these losses with policy measures such as linking a catastrophic health coverage mechanism to the Health Equity Funds, capping interest rates on health-related loans, and using loan guarantees to incentivize microfinance institutions and banks to refinance health-related, high-interest loans from money lenders. These measures would strengthen social health protection in Cambodia by enhancing financial risk protection, mitigating vulnerability to the devastating economic effects of health shocks, and reducing inequities.

### Supplementary Information

**Additional file 1. **

## Data Availability

The full data set is available upon request from the Cambodian Ministry of Planning, National Institute of Statistics.

## Footnotes

1 Cambodia does have regulations governing transparency and loan disclosure standards (including the 2017 Prakas on Resolution of Consumer Complaints).

2 The impact of Covid-19 is assessed by disaggregating the data into two time periods: before defined as the pre-Covid period (6,276 household interviews) and on or after February 15, 2020 defined as the Covid period (3,799 household interviews).
