# Supplementary material for: Hardship financing, productivity loss, and the economic cost of illness and injury in Cambodia
Source: Int J Equity Health. 2023 Oct 7;22:208. doi: 10.1186/s12939-023-02016-z (PMC10559627; doi:10.1186/s12939-023-02016-z)
Supplement: Supplementary file 1 — Additional file 1. [file 12939_2023_2016_MOESM1_ESM.docx]

**Appendices**

Appendix 1. Loan Growth in Cambodia from 2010-2018

Data source: Microfinance Information Exchange (MIX) Market

Appendix 2. Non-medical items included in consumption (extracted from the CSES questionnaire)

| **Non-food, non-medical** | **Food** |
| --- | --- |
| Clothing, footware, and apparel | Rice and other rice products |
| Transportation, accommodation, and postal | Fish, seafood and other fish products |
| Telephone and internet service, gambling, and recreation | Meat and meat products, oil or fats |
| Home equipment, maintenance, decoration, housekeeper wages, toys | Fruits or fruit products |
| Taxes on income or property | Vegetables, mushrooms, tubers and ingredients |
| Other expenses (bank payback, other financial services, tongtin, wedding gift, other gift and other contribution to other household, other expenditure) | Soft drinks, beer, wine, cigarettes, coffee... |
|  | Food and drinks away from home |
|  | Other food expenses |
|  |  |

**3 Results from Hardship Financing regression model diagnostic testing**

| **Test type** | **Test Statistic Name** | **Value** | **P-value/**  **critical value** | **Conclusion** |
| --- | --- | --- | --- | --- |
| *First Stage* | | | | |
| Underidentification | Kleibergen-Paap rank LM statistic | 102.45 | 0.0000 | Not under-identified |
|  | Sanderson-Windmeijer F test | 143.82 Chi-sq | 0.0000 | Not under-identified. |
| Weak identification | Cragg-Donald Wald F statistic | 110.89 | Stock-Yogo weak ID test critical values: 10% maximal IV size = 19.93 | Strongly identified. |
|  | Kleibergen-Paap Wald rank F statistic | 71.77 |  | Strongly identified. |
| Weak instrument-robust inference | Anderson-Rubin Wald test | 24.23  Chi-sq | 0.0000 | Strong instruments. |
| *Second Stage* | | | | |
| Underidentification | Kleibergen-Paap rank LM statistic | 102.45 | 0.0000 | Not underidentified |
| Weak identification | Cragg-Donald Wald F statistic | 110.90 | Stock-Yogo weak ID test critical values: 10% maximal IV size = 19.93 | Strongly identified. |
|  | Kleibergen-Paap rank Wald F statistic | 71.77 |  | Strongly identified. |
| Overidentification test of all instruments | Hansen J statistic | 1.16 | 0.2821 | Not overidentified. |
| Endogeneity test | Difference of two Sargan-Hansen statistics | 19.49 | 0.0000 | Total net income is endogenous. |
| Weak instrument robust test | Finlay-Magnusson-Schaffer Wald statistic | 19.90 | 0.000 | Strong instruments. |

**4.1 Results from non-medical consumption expenditure regression model diagnostic testing**

| **Test type** | **Test Statistic Name** | **Value** | **P-value/critical value** | **Conclusion** |
| --- | --- | --- | --- | --- |
| *First Stage* | | | | |
| Underidentification | Kleibergen-Paap rank LM statistic | 68.12 | 0.000 | Not under-identified |
|  | Sanderson-Windmeijer F test | 82.66  Chi-sq | 0.000 | Not under-identified. |
| Weak identification | Cragg-Donald Wald F statistic | 72.45 | Stock-Yogo weak ID test critical values: 10% maximal IV size = 19.93 | Strongly identified. |
|  | Kleibergen-Paap Wald rank F statistic | 41.24 |  | Strongly identified. |
| Weak instrument-robust inference | Anderson-Rubin Wald test | 39.46  Chi-sq | 0.000 | Strong instruments. |
| *Second Stage* | | | | |
| Underidentification | Kleibergen-Paap rank LM statistic | 68.12 | 0.0000 | Not underidentified |
| Weak identification | Cragg-Donald Wald F statistic | 72.45 | Stock-Yogo weak ID test critical values: 10% maximal IV size = 19.93 | Strongly identified. |
|  | Kleibergen-Paap rank Wald F statistic | 41.24 |  | Strongly identified. |
| Overidentification test of all instruments | Hansen J statistic | 0.40 | 0.5248 | Not overidentified. |
| Endogeneity test | Difference of two Sargan-Hansen statistics | 27.781 | 0.000 | Total net income is endogenous. |
| Weak instrument robust test | Finlay-Magnusson-Schaffer Wald statistic | 32.67 | 0.000 | Strong instruments. |

**4.2 Results from non-food, non-medical expenditure regression model diagnostic testing**

| **Test type** | **Test Statistic Name** | **Value** | **P-value/critical value** | **Conclusion** |
| --- | --- | --- | --- | --- |
| *First Stage* | | | | |
| Underidentification | Kleibergen-Paap rank LM statistic | 68.12 | 0.000 | Not under-identified |
|  | Sanderson-Windmeijer F test | 82.66  Chi-sq | 0.000 | Not under-identified. |
| Weak identification | Cragg-Donald Wald F statistic | 72.45 | Stock-Yogo weak ID test critical values: 10% maximal IV size = 19.93 | Strongly identified. |
|  | Kleibergen-Paap Wald rank F statistic | 41.24 |  | Strongly identified. |
| Weak instrument-robust inference | Anderson-Rubin Wald test | 45.08  Chi-sq | 0.000 | Strong instruments. |
| *Second Stage* | | | | |
| Underidentification | Kleibergen-Paap rank LM statistic | 68.12 | 0.000 | Not underidentified |
| Weak identification | Cragg-Donald Wald F statistic | 71.45 | Stock-Yogo weak ID test critical values: 10% maximal IV size = 19.93 | Strongly identified. |
|  | Kleibergen-Paap rank Wald F statistic | 41.24 |  | Strongly identified. |
| Overidentification test of all instruments | Hansen J statistic | 0.248 | 0.6187 | Not overidentified. |
| Endogeneity test | Difference of two Sargan-Hansen statistics | 31.39 | 0.000 | Total net income is endogenous. |
| Weak instrument robust test | Finlay-Magnusson-Schaffer Wald statistic | 38.52 | 0.000 | Strong instruments. |

**4.3 Results from food expenditure regression model diagnostic testing**

| **Test type** | **Test Statistic Name** | **Value** | **P-value/critical value** | **Conclusion** |
| --- | --- | --- | --- | --- |
| *First Stage* | | | | |
| Underidentification | Kleibergen-Paap rank LM statistic | 68.12 | 0.000 | Not under-identified |
|  | Sanderson-Windmeijer F test | 82.66  Chi-sq | 0.000 | Not under-identified. |
| Weak identification | Kleibergen-Paap Wald rank F statistic | 41.24 | Stock-Yogo weak ID test critical values: 10% maximal IV size = 19.93 | Strongly identified. |
| Weak instrument-robust inference | Anderson-Rubin Wald test | 17.48  Chi-sq | 0.0002 | Strong instruments. |
| *Second Stage* | | | | |
| Underidentification | Kleibergen-Paap rank LM statistic | 68.12 | 0.0000 | Not underidentified |
| Weak identification | Cragg-Donald Wald F statistic | 72.45 | Stock-Yogo weak ID test critical values: 10% maximal IV size = 19.93 | Strongly identified. |
|  | Kleibergen-Paap rank Wald F statistic | 41.02 |  | Strongly identified. |
| Overidentification test of all instruments | Hansen J statistic | 1.27 | 0.2598 | Not overidentified. |
| Endogeneity test | Difference of two Sargan-Hansen statistics | 11.738 | 0.0006 | Total net income is endogenous. |
| Weak instrument robust test | Finlay-Magnusson-Schaffer Wald statistic | 15.49 | 0.0001 | Strong instruments. |
